# Decreased Cytokine Plasma Levels and Changes in T-Cell Activation Are Associated With Hemodynamic Improvement and Clinical Outcomes After Percutaneous Mitral Commissurotomy in Patients With Rheumatic Mitral Stenosis

**DOI:** 10.3389/fcvm.2020.604826

**Published:** 2021-02-03

**Authors:** Vicente R. Silva, Eula G. A. Neves, Lívia S. Araújo Passos, Flávia Cristina de Melo, Andrea Teixeira-Carvalho, Maria Cecília L. Nassif, Lucas Lodi Junqueira, Elena Aikawa, Walderez O. Dutra, Maria Carmo P. Nunes

**Affiliations:** ^1^Post Graduate Program in Infectious Diseases and Tropical Medicine, School of Medicine, Universidade Federal de Minas Gerais, Belo Horizonte, Brazil; ^2^Laboratory of Cell-Cell Interactions, Department of Morphology, Institute of Biological Sciences, Universidade Federal de Minas Gerais, Belo Horizonte, Brazil; ^3^Department of Cardiovascular Medicine, The Center for Excellence in Vascular Biology, Brigham and Women's Hospital, Harvard Medical School, Boston, MA, United States; ^4^Instituto René Rachou, Fiocruz Minas, Belo Horizonte, Brazil; ^5^Serviço de Cardiologia e Cirurgia Cardiovascular do Hospital das Clínicas da Universidade Federal de Minas Gerais, Belo Horizonte, Brazil; ^6^Instituto Nacional de Ciência e Tecnologia em Doenças Tropicais (INCT-DT), Belo Horizonte, Brazil

**Keywords:** rheumatic heart disease, mitral stenosis, percutaneous mitral commissurotomy, cytokines, T cells

## Abstract

Mitral stenosis (MS) is a consequence of rheumatic heart disease that leads to heart failure requiring mechanical intervention. Percutaneous mitral commissurotomy (PMC) is the treatment of choice for the intervention, and currently there are no soluble markers associated with hemodynamic improvement after PMC. This study aims to determine the changes in cytokine/chemokine plasma levels, as well as T cell activation after PMC, and to investigate their association with immediate hemodynamic improvement and clinical outcomes. Plasma samples from eighteen patients with well-defined MS who underwent PMC and 12 healthy controls were analyzed using BioPlex immunoassay. We observed that 16 out of the 27 (60%) molecules assessed were altered in patients' plasma pre-PMC as compared to control group. Of those, IL-1β, IL-12, IL-6, IL-4, PDGF, and CCL11 showed significant decrease after PMC. Stratifying the patients according to adverse outcome after a 28-month median follow up, we detected a significant reduction of IL-1β, IL-12, IL-6, IL-4, IFN-γ, CXCL-10, VEGF, FGF and PDGF post-PMC in patients without events, but not in those who presented adverse events during the follow-up. Patients with adverse outcomes had lower IL-10 pre-PMC, as compared to the ones without adverse events. In addition, the frequency of CD8+ activated memory cells was increased after PMC, while the frequency of CD4+ activated memory cells did not change. Our results show an association between the decrease of specific cytokines and changes in T cell activation with hemodynamic improvement post-PMC, as well as with long-term outcomes, suggesting their possible use as soluble markers for hemodynamic recovery after MS intervention.

## Introduction

Rheumatic heart disease (RHD) is the major sequel of acute rheumatic fever (ARF) and is an important cause of cardiovascular mortality in children and young adults in low- and middle-income countries. There are an estimated 33 million individuals currently living with RHD, accounting for over a million premature deaths annually (1). We have demonstrated that patients with RHD have higher serum levels of inflammatory mediators than healthy controls, which is a clear evidence of ongoing inflammation (2–4). It is also known that expression of inflammatory cytokines increases in patients with significant pressure or volume overload due to valvular heart disease or chronic heart failure (5–7). Several studies have demonstrated that T-cells play an important role in the pathogenesis of RHD. It has been shown that CD4+ T cells are the main cell type found in the inflammatory infiltrates associated with damaged valves (5–7), suggesting that they play an important role in disease progression. On the other hand, a defective response of CD8+ T cells in chronic RHD patients suggests that these cells display an important immunoregulatory role in protection against RHD (8).

RHD is the major cause of mitral stenosis (MS) which is a progressive and fatal disease, if untreated. Percutaneous mitral commissurotomy (PMC) intervention is the main treatment in patients with significant MS (8–10). While it has been demonstrated that PMC decreases platelet activation and endothelial dysfunction, the alterations in plasma levels of inflammatory markers have been controversial (11–13). Currently there are no systemic markers that allow monitoring hemodynamic improvement, nor predict outcomes after PMC. We aimed to evaluate the plasma levels of cytokines pre- and post-PMC using a multiplex assay, as well as the frequency of T-cell activation using flow cytometry, in a well-defined group of severe MS patients to determine the impact of these changes on immediate results after PMC and long-term outcomes. Our results show a clear association between hemodynamic improvement and favorable outcome with a decrease in the circulating levels of IL-1β, IL-6, IL-12, IL-4 and PDGF post-PMC, as well as with changes in T-cell activation, suggesting that these measures might be useful to monitor PMC-induced hemodynamic improvement and clinical outcomes.

## Patients, Materials and Methods

### Patients

We included 18 consecutive symptomatic patients with severe rheumatic MS (mitral valve area ≤ 1.5 cm^2^) and favorable valve morphology who underwent PMC between October 17th−27th 2016 at the Federal University of Minas Gerais (UFMG) Hospital. Exclusion criteria were presence of left atrial thrombus, moderate-to-severe mitral regurgitation, concomitant severe aortic valve disease or any other contraindication to PMC. The clinical characteristics of the study population are summarized in Appendix 1. At the time of enrollment, patients had an average age of 49 ± 2 years, and 89% were females.

Echocardiographic examination was performed in all patients before and 24 h after the procedure. PMC was performed according to the antegrade, trans-septal technique with Inoue balloon catheter (Toray Medical Corporation; Tokyo, Japan).

The control group was composed of 12 healthy volunteers. Peripheral blood samples were collected immediately before PMC, and 4–11 days after the procedure. The date of enrollment in the study was defined as the date on which PMC was performed. Adverse outcome was defined as the need for mitral valve intervention, either percutaneous or surgical, death related to MS, or new onset of atrial fibrillation. Patients with event-free during the follow-up were considered with favorable outcome. Informed consent was obtained from all participants and the UFMG ethical review board approved the protocol. The work described has been carried out in accordance with The Code of Ethics of the World Medical Association (Declaration of Helsinki).

### Measurement of Plasma Cytokines, Chemokines, and Growth Factors

Plasma concentrations of cytokines/chemokines/growth factors were measured using Luminex immunoassay kit (Bio-Plex Pro™ Human Cytokine 27-plex Assay, Bio-Rad, Hercules—CA, USA). Results were presented as mean fluorescence intensity (MFI).

### Analysis of Frequency of T-cell Activation by Flow Cytometry

Purification of peripheral blood mononuclear cells (PBMC) and immunofluorescence staining was performed as previously described (14). Briefly, heparinized blood was applied over a Ficoll-Hypaque (GE Healthcare Life Sciences) gradient, centrifuged at 600 g for 40 min, at room temperature, and PBMC were collected at the interface between the plasma and Ficoll. Cells were washed 3 times by centrifugation with PBS and resuspended in PBS at a concentration of 10^7^ cells/ml. Cells were plated at 2 × 10^5^ cells/well in a 96 U-bottom plate, incubated with a 40 μL mix of monoclonal antibodies CD4-Percp-Cy5.5 (clone OKT4, 1:20 dilution), anti-CD8-APCCy7 (clone SK1, 1:20 dilution), anti-CD45RO-APC (clone UCHL1, 1:40 dilution) and anti-CD69-PECy7 (clone FN50, 1:20 dilution) for 15 min at 4°C, washed with PBS, and fixed for 20 min with 2% formaldehyde. Samples were acquired on the FACS CANTO II and analyzed using FlowJo software (Tree Star, Ashland, OR, USA). Gating strategy was performed selecting the lymphocyte population in a forward vs. side scater graph (**Figure 3A**). Further gating on CD4+ (**Figure 3B**) or CD8+ cells (**Figure 3C**) were performed, and the expression of CD69 and CD45RO were concomitantly evaluated in the gated CD4+ and CD8+ cells, to determine the frequency of activated cells (**Figures 3B,C** for CD4 and CD8, respectively). All antibodies were obtained from BioLegend (San Diego, CA, USA).

### Gene Co-expression Network for Human Immune System Cell Types

Co-expression network was constructed through direct and indirect relationships of CD8+ T cells and plasma soluble factors measured by Luminex immunoassay. Pairwise correlation prediction was determined based on Immuno-Navigator (sysimm.ifrec.osaka-u.ac.jp/immuno-navigator/) and network constructed using NetworkAnalyst.ca. Resulting high-scoring genes that display high correlation of expression in T CD8-derived genes were used to identify hub genes and functional enrichment pathways. Regulatory interactions emerging as a result of interconnections in the network were generated using Kyoto Encyclopedia of Genes and Genomes (KEGG).

### Statistical Analysis

The sample size was performed to achieve 80% power for detecting a reduction of up to 30% in IL-6 concentration after PMC with a two-sided type-I error rate of 5% (2). Statistical analyses were performed using SPSS™ software version 25 for Windows (SPSS Inc., Chicago—IL, USA) and GraphPad Prism™ version 7.00 for Windows (GraphPad Software, La Jolla—CA, USA). Continuous variables were expressed as mean ± standard deviation (SD) or as median and interquartile range when appropriate. Categorical variables were expressed as frequency and percentage. We used the Mann-Whitney test to access differences between study group and control group. Wilcoxon signed-rank test was used to evaluate differences within the study group pre and post-PMC. A two-tailed *P*-value of < 0.05 was considered to be statistically significant.

## Results and Discussion

**Figure 1:**
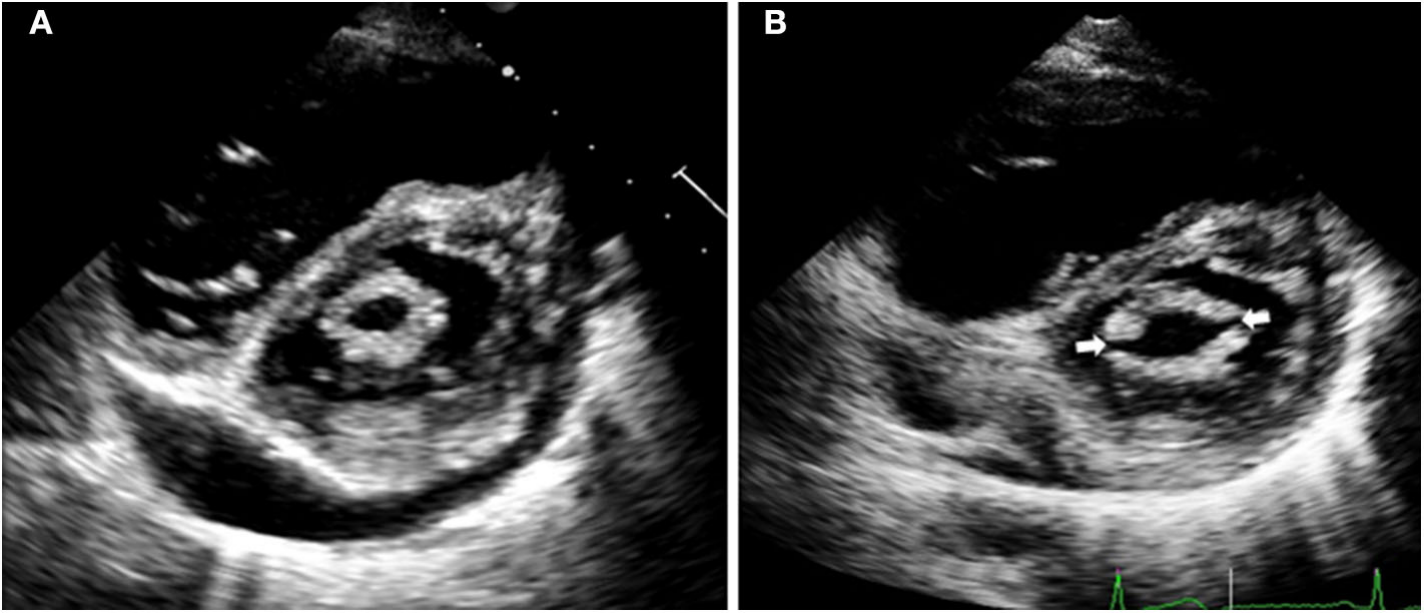
Echocardiographic images of a patient with severe mitral stenosis before percutaneous mitral commissurotomy **(A)**. After the procedure **(B)**, both commissures are open (arrows), which increases the mitral valve area resulting in immediate hemodynamic improvement.

To assess the hemodynamic improvement after PMC, valve area, transmitral gradient, left atrial pressure and pulmonary pressure were measured before and immediately after the procedure. After PMC, mitral valve are increased from 1.06 ± 0.2 to 1.90 ± 0.3 cm^2^ (*p* = 0.001) (Figure 1), and transmitral mean gradient decrease from 9.7 ± 4.3 to 4.6 ± 1.2 mmHg (*p* = 0.009). Similarly, left atrial pressure invasively measured decrease from 19.6 ± 4.4 to 17.1 ± 4.3 mmHg (*p* = 0.007), and mean pulmonary pressure decrease from 23.2 ± 3.9 to 21.2 ± 5.2 mmHg (*p* = 0.007).

Plasma levels of soluble molecules in control group and in MS group pre- and post-PMC are presented in Appendix 2–4.

First we compared the levels of growth factors, chemokines and cytokines between control group and MS patients pre-PMC (Appendix 2). We observed that there were no significant differences in levels of growth factors (GM-CSF, G-CSF, VEGF, FGF) and proliferative cytokines (IL-2, IL-7, IL-15) among MS group and control group, except for PDGF, that was significantly higher in patients pre-PMC (Appendix 2). We attribute de absence of difference in levels of growth factors and proliferative factors among patients and controls to the late chronic stage of the disease. Levels of all chemokines measured were higher in MS patients pre-PMC than control group, possibly related to ongoing inflammation (Appendix 2). Levels of TNF-α, IL-10, IL-5, and IL-9 did not change comparing both groups (Appendix 2). However, levels of IL-1β, IL-1RA, IL-4, IFN-γ, and IL-17 were higher in MS pre-PMC compared to control group (Appendix 2). The higher levels of inflammatory cytokines IL-1β, IL-17, and IFN-γ in MS compared to control group are suggestive of the ongoing inflammation. In particular, IL-17 is a potent cytokine related to autoimmune diseases, and previously associated with severity of RHD (12). The high levels of down-regulatory cytokines IL-1RA and IL-4 suggest that these cytokines may attempt to counterbalance inflammation in this phase of disease. Interestingly, levels of IL-12p70, IL-13 and IL-6 were lower in MS group as compared to controls (Appendix 2).

We then compared the levels of circulating growth factors, chemokines and cytokines between MS patients pre-PMC and post-PMC (Appendix 3). Comparing levels of growth factors and proliferative cytokines in patients pre- and post-PMC, we demonstrated that while the levels of GM-CSF and G-CSF did not change (Appendix 3), a decrease in plasma levels of PDGF, VEGF, FGF basic, IL-2, IL-7 and IL-15 was observed post-PMC (Appendix 3), probably due to hemodynamic improvement. Of note, levels of VEGF were negatively correlated with mitral valve area (*p* = 0.052, *r*^2^ = 0.03). Rajamannan et al. (11) hypothesized that the mechanism of valvular calcification included neoangiogenesis in valve tissue and demonstrated the presence of VEGF in rheumatic valves. Evaluating the levels of chemokines, only CCL11 decrease in patients post-PMC as compared to pre-PMC (Appendix 3). Given CCL11 is involved in recruitment of polymorphonuclear cells, especially neutrophils, which are the first cells to respond in inflammatory settings, it is reasonable to think that the hemodynamic improvement due to adequate relief of valve obstruction may have a prompt neutrophil response. In addition, given the observed decrease in CCL11, IL-4 and IL-5 post-PMC, it is possible to hypothesize that PMC decreases activation of polymorphonuclear cells, high producers of these cytokines. Indeed, there were strong correlations between levels of CCL11 and both IL-4 (*r* = 0.87) and IL-5 (*r* = 0.88). Evaluating the levels of cytokines, we observed that the levels of mostly T cell-derived inflammatory cytokines (IL-17, IFN-γ) did not change comparing post- and pre-PMC (Appendix 3). Post-PMC, levels of IL-1β, IL-12p70, IL-6, IL-4, and IL-5 had a significant decrease, as compared to pre-PMC (Appendix 3). Consistent with our findings, previous studies have demonstrated that levels of IL-6 and TNF-α decrease in this phase of the disease (13, 14). Of note, the levels of IL-12, IL-1β, and IL-6, inflammatory cytokines produced primarily by monocytes/macrophages and related to innate response, were decreased. Improvement of hemodynamic parameters after PMC may be responsible for this decrease, and the improvement might interfere first with innate immune response and later in adaptive response. We expected a decrease in levels of TNF-α and IFN-γ, as demonstrated by Cagli et al., who showed that TNF-α progressively decreased at the 24th h until the 4th week after PMC (15). However, Cagli *et al* evaluated only patients in sinus rhythm and with no other comorbidity; therefore, it is possible that, in our group, the levels of TNF-α follow a different kinetics than the one observed by those authors. The fact that levels of down-regulatory cytokines IL-10 and IL-1RA did not change after PMC (Appendix 3) is probably associated with the reduction in inflammation observed post-PMC. The lower levels of IL-10 pre-PMC were associated with adverse outcomes (No adverse outcome IL-10 pre-PMC = 16, and adverse outcome IL-10 pre-PMC = 14; *p* = 0.04), emphasizing the role of IL-10 in controlling inflammation and possibly preventing restenosis over time.

Finally, we then compared the levels of growth factors, chemokines and cytokines between control group and MS patients post-PMC (Appendix 4). PDGF was the only molecule among growth factors and proliferative cytokines that was higher in patients pre-PMC compared to controls. After PMC, although its levels have fallen, they were still higher compared to controls (Appendix 4). On the other hand, while all the chemokines presented higher plasma levels in patients pre-PMC compared to controls and only CCL11 had a significant decrease in plasma levels post-PMC, the levels of CXCL10, CCL11, and IL-8 were not statistically different from controls (Appendix 4). IL-5 and IL-1β, that presented a significant decrease after PMC, demonstrated levels post-PMC statistically not different from controls, but IL-4, that also demonstrated significant decrease after PMC, still presented higher levels than controls (Appendix 4). Levels of IL-17, IFN-γ, and IL-1RA continued statistically higher post-PMC compared to controls (Appendix 4). IL-10 levels were not different from controls neither pre nor post-PMC (Appendix 4). Interesting, levels of TNF-α, IL-9, IL-6, IL-12p70, and IL-13 were lower in patients post-PMC compared to controls (Appendix 4).

**Figure 2:**
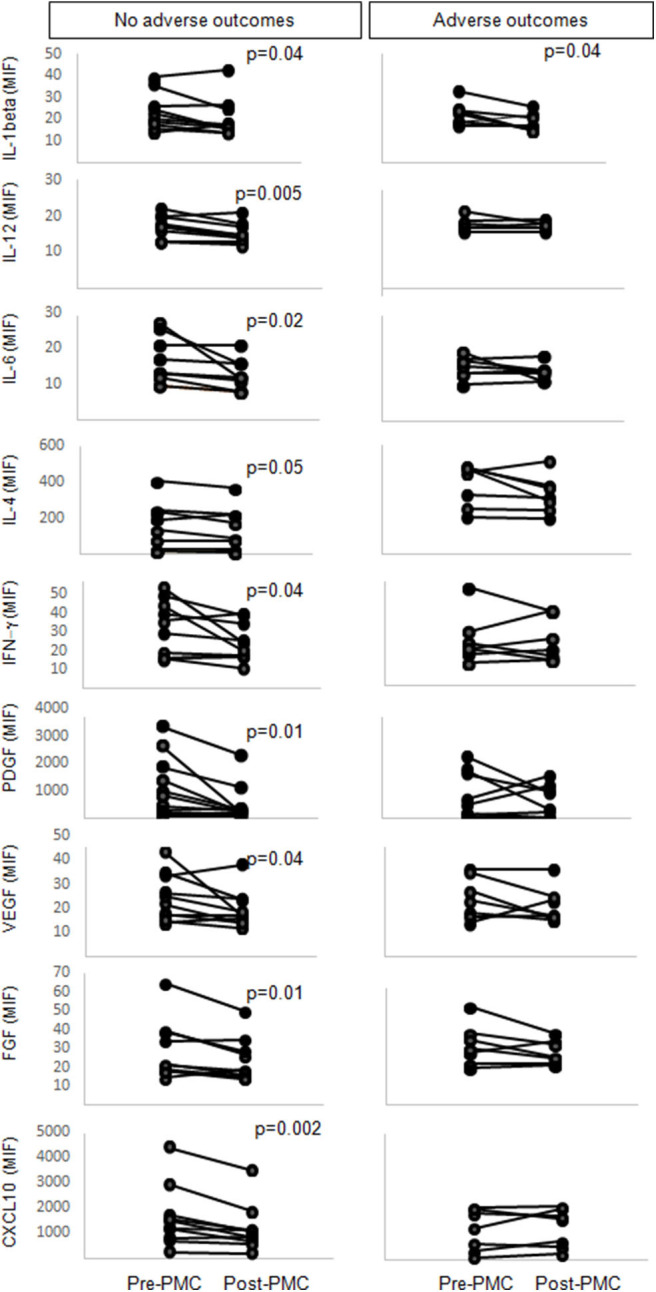
Levels of IL-1β, IL-12, IL-6, IL-4, IFN-γ, PDGF, VEGF, FGF-basic and CXCL10 in plasma from mitral stenosis patients pre- and post- percutaneous mitral commissurotomy (PMC). Patients were followed-up for a median of 28 months and categorized in groups without or with adverse outcomes. Statistically significant differences between levels of soluble factors pre- and post-PMC are indicated.

We then stratified the patients according to clinical outcomes at long-term follow-up and compared the levels of soluble factors pre- and post-PMC in those groups. During a median follow-up of 28 months (range, 4–40), 10 patients (55.5%) were clinically stable, asymptomatic, whereas 8 (44.4%) patients presented adverse outcomes, including five mitral valve replacements, one repeat PMC, and two onset of atrial fibrillation. The analysis showed that levels of IL-1β decreased in patients with and without adverse outcomes (Figure 2), while levels of IL-12, IL-6, IL-4, IFN-γ, PDGF, VEGF, FGF, CXCL10 were decreased only in patients who did not display adverse events, post-PMC as compared to pre-PMC (Figure 2). This decrease was not significant in patients with adverse events (Figure 2). All these molecules, except for CXCL-10, presented decreased levels post-PMC, even before considering PMC outcome after follow-up, reinforcing their possible usefulness as markers of PMC-induced hemodynamic improvement and favorable outcome.

**Figure 3:**
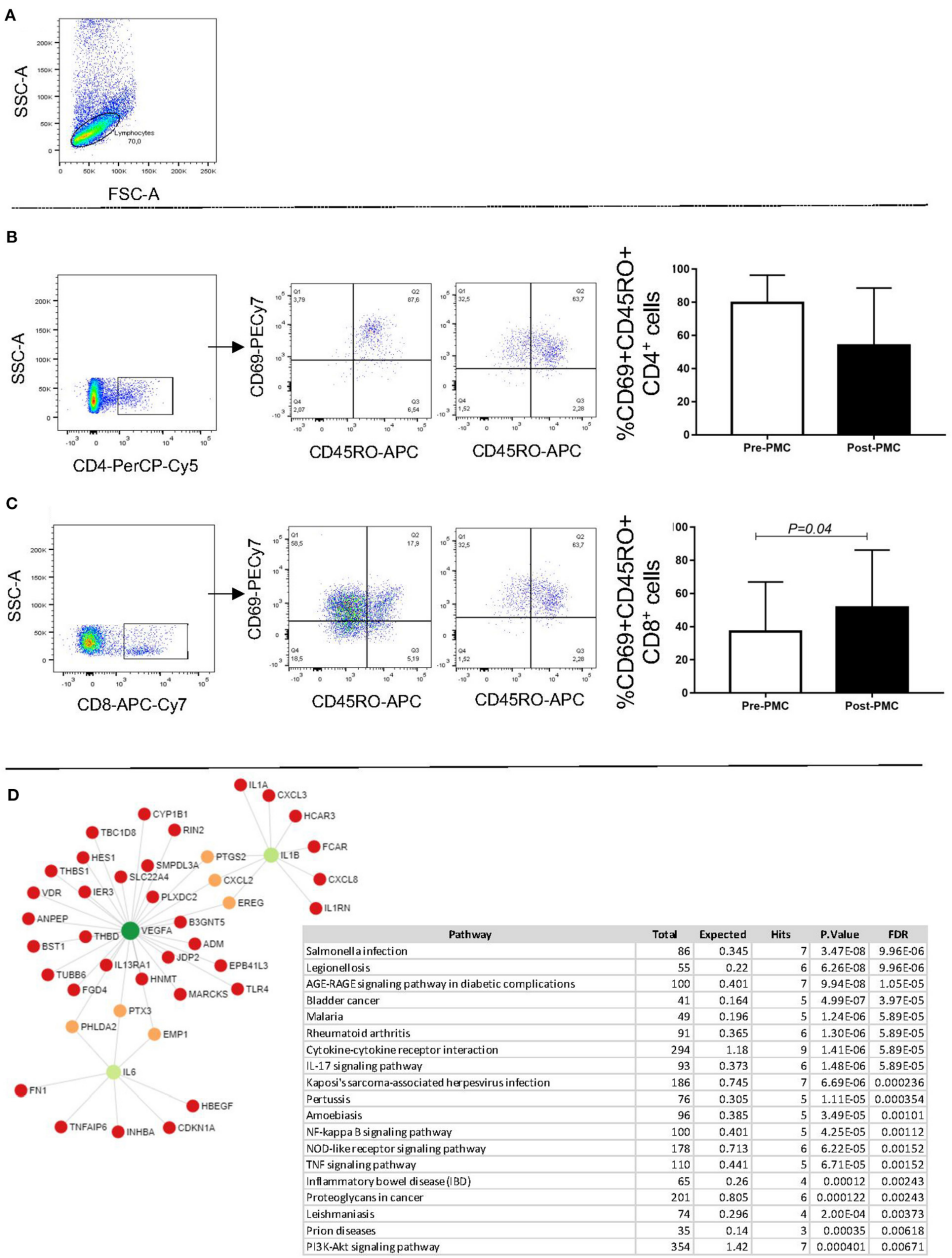
Analysis of the percentage of circulating CD4+CD45RO+CD69+ and CD8+ CD45RO+CD69+ T cells pre and post PMC, and network interactions. PBMC were obtained from the patients before and after PMC, and processed for flow cytometry analysis, as described in Material and Methods. **(A)** Shows a representative dot plot with the selection of the lymphocyte gate. **(B)** Shows the selection of CD4+ T cells, representative dot lots showing the frequency of CD69+CD45RO+ cells, and bar graphs for the analysis before (white bars) and after (black bars) PMC. **(C)** Shows the selection of CD8+ T cells, representative dot lots showing the frequency of CD69+CD45RO+ cells, and bar graphs for the analysis before (white bars) and after (black bars) PMC. Data are expressed as average and standard deviation, and statistical significance is indicated by the *p*-value. **(D)** Co-expression network of soluble factors overrepresented in plasma from patients without adverse outcomes in a background of T CD8-derived genes. KEEG enriched pathways analysis obtained from gene co-expression network.

In order to determine the influence of PMC on T cell activation, we evaluated the expression of CD69 by CD4+CD45RO+ and CD8+CD45RO+ T cells. The expression of CD45RO is associated with pre-activated/memory cells (16). Our data showed that, while not statistically significant, there was a slight reduction in the frequency of CD4+CD45RO+CD69+ cells post-PMC (Figure 3). On the other hand, the percentage of CD8+CD45RO+CD69+ cells significantly increased after PMC (Figure 3). The observed increase in a pre-activated/memory CD8+ T cell population, previously associated with immunoregulatory functions in RHD (8), together with a tendency to a reduction in the frequency of memory/activated CD4+ cells associated with pathology in RHD (5–7), might reflect the clinical amelioration after PMC. Further studies to determine the functional characteristics of these cells before and after PMC will provide important additional information on their role in MS.

To extend the findings, we then turned to the Immuno-Navigator database to verify co-expression between T CD8-derived genes and the nine altered soluble factors in patients without adverse outcomes. VEGF, IL-1beta and IL-6 were the highly connected genes in a T CD8 co-expression network and therefore might be more relevant to the functionality than other nodes in the network. However, the network analysis shows that VEGF, IL-1β and IL-6 display direct influence in several other genes. Thus, the observed decrease in the levels of these factors after successful PMC will also interfere with the overall inflammatory response. The top 20 KEGG enrichment analyses identified common pathways of infectious diseases, as well as an autoimmune disease, rheumatoid arthritis. Among all enriched inflammatory pathways, we found AGE-RAGE, IL-17, NF-kappa B, NOD-like receptor and TNF signaling pathways, which are under the influence of the three identified hub genes. This entices future studies to further investigate the role of these node molecules as biomarkers and/or potential intervention candidates.

PMC is a safe and effective procedure for treating patients with MS, which results in the alleviation of valve obstruction with hemodynamic and symptoms improvement. Despite its wide use, there are currently no soluble markers to evaluate PMC-induced hemodynamic improvement, nor clinical outcomes. Using cytokine levels to assess outcome of percutaneous coronary intervention has shown promising results (17). Our study is the first to evaluate the impact of PMC on plasma levels of a broader range of cytokines, chemokines and growth factors, together with the frequency of activated circulating T cell subpopulations. Importantly, we showed a clear association between the levels of particular circulating molecules with hemodynamic improvement immediately after PMC, as well as with clinical outcomes. These data suggest useful immunologic prognostic markers of amelioration after PMC and at long term follow up.

## Data Availability Statement

All datasets presented in this study are included in the article.

## Ethics Statement

The studies involving human participants were reviewed and approved by Federal University of Minas Gerais UFMG - Ethical Review Board. The patients/participants provided their written informed consent to participate in this study.

## Author Contributions

VS: patient care, procedure, and follow up and data analysis and writing. EN: experimentation and data preparation. LP: data analysis and methodology. FC: material collection and experimentation. AT-C: supervision of experimentation. MN: patient care and follow up. LJ: patient care and procedure. EA: bioinformatic analysis and critical revision of the manuscript. WD: conceptualization, data analysis and interpretation, and writing. MN: conceptualization, data analysis and interpretation, patient care, and writing. All authors contributed to the article and approved the submitted version.

## Conflict of Interest

The authors declare that the research was conducted in the absence of any commercial or financial relationships that could be construed as a potential conflict of interest.

## Figures and Tables

**Appendix 1:** Baseline characteristics of the study population.

**Characteristics**	**Mitral stenosis group**	**Control group**
	**(*n* = 18)**	**(*n* = 12)**
Age (years)	49.28 ± 2.45	17.25 ± 0.13
Female sex, n (%)	16 (88.9%)	4 (33.3%)
Age at diagnostic (years)	35.6 ± 3.28	–
**Functional class NYHA:**
I and II, *n* (%)	12 (66.7%)	–
III and IV, *n* (%)	6 (33.3%)	–
Wilkins score	7 (7–8)	–
Atrial fibrillation, *n* (%)	8 (44.4%)	–
Prior embolic events, *n* (%)	2 (11.1%)	–
Heart failure at admission, *n* (%)	9 (50.0%)	–
Diabetes mellitus, *n* (%)	4 (22.2%)	–
Hypertension, *n* (%)	8 (44.4%)	–
Prior valve intervention, *n* (%)	5 (27.8%)	–
Diuretics, *n* (%)	14 (77.8%)	–
Beta-blockers, *n* (%)	14 (77.8%)	–
Digital, *n* (%)	2 (11.1%)	–
Warfarin, *n* (%)	6 (33.3%)	–
Aspirin, *n* (%)	2 (11.1%)	–
**Antibiotic prophylaxis**
Current, *n* (%)	4 (22.2%)	–
Previous, *n* (%)	10 (55.6%)	–

*Data are presented as mean ± SD or absolute numbers (percentage)*.

*MS, mitral stenosis; NYHA, New York Heart Association*.

**Appendix 2:** Levels of soluble factors in control group and study group pre-PMC.

**Variables**	**Control group**	**Pre-PMC group**	***P*-value**
**Growth factors**
GM-CSF	18 (15–19)	17 (13–21)	0.55
G-CSF	33 (30–42)	51 (33–77)	0.10
VEGF	22 (21–27)	23 (17–34)	0.84
FGF basic	20 (18–21)	24 (19–37)	0.10
**PDGF**	**124 (50–193)**	**820 (199–1,810)**	**0.001**
**Proliferative factors**
IL-2	16 (15–17)	17 (13-23)	0.53
IL-7	23 (22–24)	22 (20–23)	0.57
IL-15	24 (23–26)	20 (19–27)	0.11
**Chemokines**
**CXCL10**	**685 (365–1,104)**	**1,280 (690–1,971)**	**0.03**
**CCL2**	**45 (24–54)**	**171 (69–357)**	**<0.001**
**CCL3**	**33 (25–46)**	**375 (98–6,394)**	**<0.001**
**CCL4**	**329 (318–441)**	**1,265 (736–2,304)**	**<0.001**
**CCL11**	**520 (454–925)**	**2,533 (448–3,614)**	**0.03**
**CCL5**	**1,662 (1,427–1,824)**	**2,574 (1,738–2,995)**	**0.02**
**IL-8**	**28 (26–36)**	**65 (33–467)**	**0.01**
**Cytokines**
TNF-α	28 (24–37)	22 (16–31)	0.13
IL-10	18 (16–22)	16 (13–22)	0.09
IL-5	16 (14–20)	18 (14–24)	0.37
IL-9	47 (43–49)	42 (34–49)	0.18
**IL-1** β	**17 (16–18)**	**23 (18–26)**	**0.01**
**IL-6**	**19 (18–20)**	**13 (13–18)**	**0.02**
**IL-12p70**	**18 (17–25)**	**16 (14–18)**	**0.01**
**IL-17**	**26 (24–29)**	**62 (29–93)**	**<0.001**
**IFN-γ**	**16 (13–16)**	**23 (17–42)**	**<0.001**
**IL-1ra**	**21 (21–25)**	**270 (155–1,057)**	**<0.001**
**IL-4**	**29 (23–34)**	**136 (49–178)**	**0.003**
**IL-13**	**19 (17–19)**	**15 (13–18)**	**0.01**

*Data are presented as median and interquartile range (Q1–Q3). PMC, percutaneous mitral commissurotomy. Bold values represent statistically significant differences*.

**Appendix 3:** Levels of soluble factors in study group pre and post-PMC.

**Variables**	**Pre-PMC group**	**Post-PMC group**	***P*-value**
**Growth factors**
GM-CSF	17 (13–21)	14 (12–18)	0.06
G-CSF	51 (33–77)	39 (31–56)	0.14
**VEGF**	**23 (17–34)**	**17 (16–24)**	**0.03**
**FGF** basic	**24 (19–37)**	**22 (17–29)**	**0.01**
**PDGF**	**820 (199–1,810)**	**314 (210–1,033)**	**0.02**
**Proliferative factors**
**IL-2**	**17 (13–23)**	**14 (13–19)**	**0.01**
**IL-7**	**22 (20–23)**	**19 (18–23)**	**0.03**
**IL-15**	**20 (19–27)**	**20 (18–27)**	**0.03**
**Chemokines**
CXCL10	1,280 (690–1,971)	1,088 (625–1,815)	0.05
CCL2	171 (69–357)	127 (48–324)	0.20
CCL3	375 (98–6,394)	212 (83–1,513)	0.06
CCL4	1,265 (736–2,304)	1,018 (576–1,586)	0.16
**CCL11**	**2,533 (448–3,614)**	**1,966 (301–2,971)**	**0.01**
CCL5	2,574 (1,738–2,995)	2,377 (1,732–3,004)	0.40
IL-8	65 (33–467)	29 (25–58)	0.14
**Cytokines**
TNF-α	22 (16–31)	19 (14–27)	0.10
IL-10	16 (13–22)	14 (12–24)	0.88
**IL-5**	**18 (14–24)**	**15 (13–20)**	**0.01**
IL-9	42 (34–49)	35 (30–40)	0.10
**IL-1** **β**	**23 (18–26)**	**17 (16–24)**	**0.02**
**IL-6**	**13 (13–18)**	**12 (11–15)**	**0.004**
**IL-12p70**	**16 (14–18)**	**14 (14–16)**	**0.01**
IL-17	62 (29–93)	40 (31–73)	0.06
IFN-γ	23 (17–42)	21 (17–37)	0.13
IL-1ra	270 (155–1,057)	282 (143–654)	0.31
**IL-4**	**136 (49–178)**	**99 (49–176)**	**0.01**
IL-13	15 (13–18)	14 (13–16)	0.09

*Data are presented as median and interquartile range (Q1–Q3). PMC, percutaneous mitral commissurotomy. Bold values represent statistically significant differences*.

**Appendix 4:** Levels of soluble factors in control group and study group post-PMC.

**Variables**	**Control group**	**Post-PMC group**	***P*-value**
**Growth factors**
**GM-CSF**	**18 (15–19)**	**14 (12–18)**	**0.02**
G-CSF	33 (30–42)	39 (31–56)	0.40
**VEGF**	**22 (21–27)**	**17 (16–24)**	**0.03**
FGF basic	20 (18–21)	22 (17–29)	0.56
**PDGF**	**124 (50–193)**	**314 (210–1,033)**	**0.002**
**Proliferative factors**
IL-2	16 (15–17)	14 (13–19)	0.09
**IL-7**	**23 (22–24)**	**19 (18–23)**	**0.03**
**IL-15**	**24 (23–26)**	**20 (18–27)**	**<0.001**
**Chemokines**
CXCL10	685 (365–1,104)	1,088 (625–1,815)	0.10
**CCL2**	**45 (24–54)**	**127 (48–324)**	**0.002**
**CCL3**	**33 (25–46)**	**212 (83–1,513)**	**<0.001**
**CCL4**	**329 (318–441)**	**1,018 (576–1,586)**	**<0.001**
CCL11	520 (454–925)	1,966 (301–2,971)	0.06
**CCL5**	**1,662 (1,427–1,824)**	**2,377 (1,732–3,004)**	**0.04**
IL-8	28 (26–36)	29 (25–58)	0.50
**Cytokines**
**TNF-α**	**28 (24–37)**	**19 (14–27)**	**0.01**
IL-10	18 (16–22)	14 (12–24)	0.08
IL-5	16 (14–20)	15 (13–20)	0.61
**IL-9**	**47 (43–49)**	**35 (30–40)**	**<0.001**
IL-1 β	17 (16–18)	17 (16–24)	0.72
**IL-6**	**19 (18–20)**	**12 (11–15)**	**<0.001**
**IL-12p70**	**18 (17–25)**	**14 (14–16)**	**0.01**
**IL-17**	**26 (24–29)**	**40 (31–73)**	**0.01**
**IFN-γ**	**16 (13–16)**	**21 (17–37)**	**0.001**
**IL-1ra**	**21 (21–25)**	**282 (143–654)**	**<0.001**
**IL-4**	**29 (23–34)**	**99 (49–176)**	**0.01**
**IL-13**	**19 (17–19)**	**14 (13–16)**	**<0.001**

*Data are presented as median and interquartile range (Q1–Q3). PMC, percutaneous mitral commissurotomy. Bold values represent statistically significant differences*.
